# Exploiting the Yeast L-A Viral Capsid for the *In Vivo* Assembly of Chimeric VLPs as Platform in Vaccine Development and Foreign Protein Expression

**DOI:** 10.1371/journal.pone.0000415

**Published:** 2007-05-02

**Authors:** Frank Powilleit, Tanja Breinig, Manfred J. Schmitt

**Affiliations:** 1 Angewandte Molekularbiologie, Universität des Saarlandes, Saarbrücken, Germany; 2 Medizinische Mikrobiologie und Hygiene, Institut für Virologie, Universität des Saarlandes, Homburg, Germany; University of Geneva, Switzerland

## Abstract

A novel expression system based on engineered variants of the yeast (*Saccharomyces cerevisiae*) dsRNA virus L-A was developed allowing the *in vivo* assembly of chimeric virus-like particles (VLPs) as a unique platform for a wide range of applications. We show that polypeptides fused to the viral capsid protein Gag self-assemble into isometric VLP chimeras carrying their cargo inside the capsid, thereby not only effectively preventing proteolytic degradation in the host cell cytosol, but also allowing the expression of a *per se* cytotoxic protein. Carboxyterminal extension of Gag by T cell epitopes from human cytomegalovirus pp65 resulted in the formation of hybrid VLPs that strongly activated antigen-specific CD8^+^ memory T cells *ex vivo*. Besides being a carrier for polypeptides inducing antigen-specific immune responses *in vivo*, VLP chimeras were also shown to be effective in the expression and purification of (i) a heterologous model protein (GFP), (ii) a *per se* toxic protein (K28 α-subunit), and (iii) a particle-associated and fully recyclable biotechnologically relevant enzyme (esterase A). Thus, yeast viral Gag represents a unique platform for the *in vivo* assembly of chimeric VLPs, equally attractive and useful in vaccine development and recombinant protein production.

## Introduction

Viral expression systems can be classified into three types based on the regulatory and/or structural viral component that drives protein expression: (i) plasmid-based vectors containing promoter elements from either pro- or eukaryotic viruses; (ii) infectious viral vectors in which the gene of interest is integrated into the viral genome and expressed from a viral promoter in an appropriate host; (iii) virus-like particles (VLPs), also called pseudovirions, representing subunit structures composed of multiple copies of a viral capsid and/or envelope protein capable to self-assemble into VLPs of defined spherical symmetry *in vivo* [1]–[3]. Currently, VLPs composed of a structural protein are often used as particulate antigen in the design of prototype vaccines as they possess several advantages over conventional monomeric protein immunogens [4]. Firstly, most VLPs can be produced in large quantity in a heterologous host. Secondly, due to their particle structure and high molecular weight, VLPs can be easily purified in a preparative scale. Thirdly, a number of particle forming proteins tolerate insertion of foreign amino acid sequences without affecting *in vivo* self-assembly competence. Such chimeric or hybrid VLPs, exploited as platform for the display of antigenic determinants in a polyvalent manner, have already been shown to be promising candidates in the development of various subunit vaccines [5].

Here, a novel expression system based on the non-infectious yeast (*S. cerevisiae*) dsRNA virus L-A was designed. This mycovirus represents an autonomously replicating, encapsidated dsRNA element that stably persists in the cytoplasm of an infected yeast cell without conferring a recognizable phenotype upon its host [6]. As member of the *Totiviridae* family, L-A contains a linear non-segmented dsRNA genome (4.6 kb) comprising two overlapping ORFs, *gag* and *pol*. While *gag* encodes the major capsid protein Gag (76 kDa), *pol* specifies a multifunctional RDRP which is *in vivo* expressed as a 171 kDa Gag/Pol fusion protein by a [−1] ribosomal frame-shift event [6], [7]. As Gag has been shown to be sufficient to drive *in vivo* self-assembly into VLPs, Pol is dispensable for viral coat assembly [8]. However, N-acetylation of Gag (catalyzed by Mak3p of the host cell) is an essential prerequisite for VLP formation *in vivo* [9]. The 40 nm L-A capsid has a 120-subunit structure composed of 118 Gag proteins and two copies of Gag/Pol configured as an icosahedron of triangulation class T = 1 [7], [10]–[12]. In the present study, we used Gag - and specifically designed variants thereof - for the *in vivo* assembly of VLP chimeras suitable for heterologous protein production and display of vaccine-relevant immunogens.

## Results

### Chimeric Gag assembles into yeast VLPs

**Figure 1:**
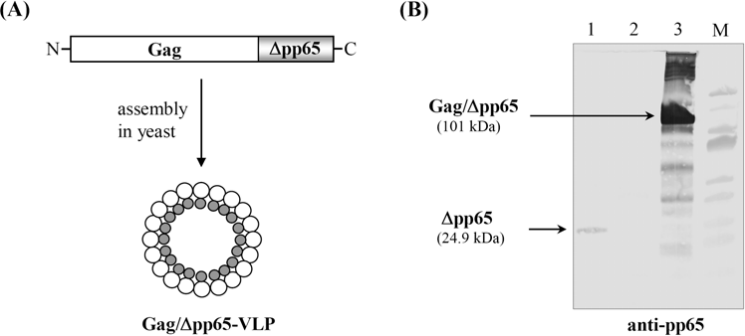
Expression of a Gag/Δpp65 fusion protein in yeast. (A) Schematic outline of Gag/Δpp65 before and after *in vivo* assembly into chimeric yeast VLPs. (B) SDS-PAGE and anti-pp65 immunoblot of crude extracts from yeast expressing either Δpp65 (lane 1), Gag (lane 2), or Gag/Δpp65 (lane 3). To ensure *in vivo* translation initiation of N-terminally truncated Δpp65 (24.9 kDa), a methionine residue was added to the N-terminus of Δpp65 [M, prestained PAGE Ruler, Fermentas].

**Figure 2:**
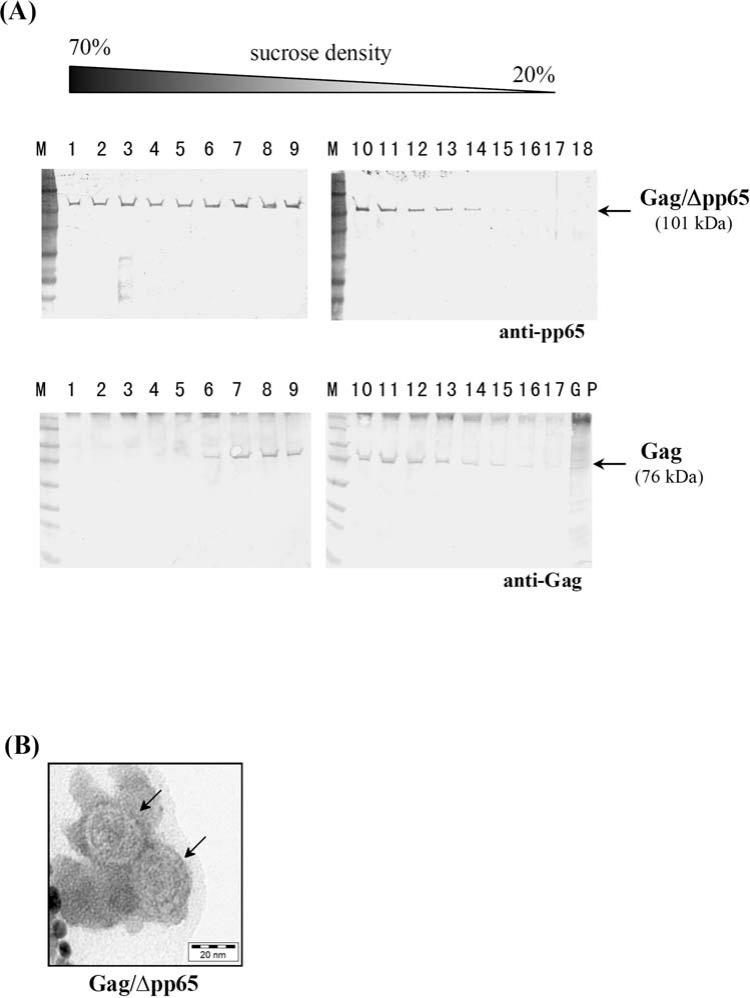
Sedimentation profile and electron microscopy of Gag/Δpp65 expressed in yeast demonstrate *in vivo* assembly into isometric VLP chimeras. (A) Western analysis of chimeric Gag/Δpp65 particles and natural L-A virions assembled in yeast and purified by ultracentrifugation through a linear sucrose gradient. Aliquots of each gradient fraction were separated by SDS-PAGE and probed with monoclonal anti-pp65 and polyclonal anti-Gag, respectively [GP, gradient pellet; M, full range rainbow marker, Amersham]. (B) Electron micrograph of sucrose-gradient-purified Gag/Δpp65 after negative staining with uranyl acetate/methyl cellulose [magnification 150,000; arrows indicate Gag/Δpp65 particles].

Since in the natural L-A virus, Pol (as C-terminal part of Gag/Pol) extends into the interior of the capsid to ensure replication and transcription of the viral genome [11], we replaced Pol by a truncated version of the immunodominant phosphoprotein pp65 from human cytomegalovirus (HCMV) to modify the inner surface of the capsid. The truncated protein (Δpp65) comprised the C-terminal amino acids 358-561 of pp65 flanked by the CD8^+^ T-cell epitopes AE44 and AE45 [13] at its N- and C-terminus, respectively. The resulting Gag/Δpp65 protein fusion (101 kDa) as well as non-modified (naked) Δpp65 (24.9 kDa) were separately expressed in yeast and analyzed for expression level and protein stability. In the Gag/Δpp65 protein fusion, Δpp65 is fused in the [0]-frame to the 3′-end of *gag* resulting in a protein fusion that is ought to self-assemble (*via* its Gag domain) into VLPs encapsulating Δpp65 as C-terminal cargo (Figure 1A). Western analysis of cell extracts from yeast expressing either naked Δpp65 or Gag/Δpp65 revealed only a weak signal for non-fused Δpp65 in contrast to an intense signal seen in cells expressing Gag/Δpp65 (Figure 1B). The observed instability of the naturally short-lived Δpp65 protein in the multiple protease-deficient mutant strain S86c could not even be prevented in mutant hosts defective in components of the ubiquitin-proteasome-system (UPS) nor in a yeast Δ*pep4* mutant devoid of vacuolar proteases (data not shown). Interestingly however, Δpp65 was significantly stabilized and effectively protected from proteolytic degradation when expressed in a particulate manner as C-terminal protein fusion to Gag (Figure 1B). The competence of Gag/Δpp65 for *in vivo* self-assembly into hybrid VLPs was demonstrated by analyzing its sedimentation profile during sucrose gradient centrifugation and by electron microscopy of gradient-purified VLPs: Gag/Δpp65 formed isometric particles which showed a similar sedimentation behaviour as natural L-A virions (Figure 2A and 2B).

### Gag/Δpp65 activates HCMV-specific CD8^+^ memory T-cells *ex vivo*

**Figure 3:**
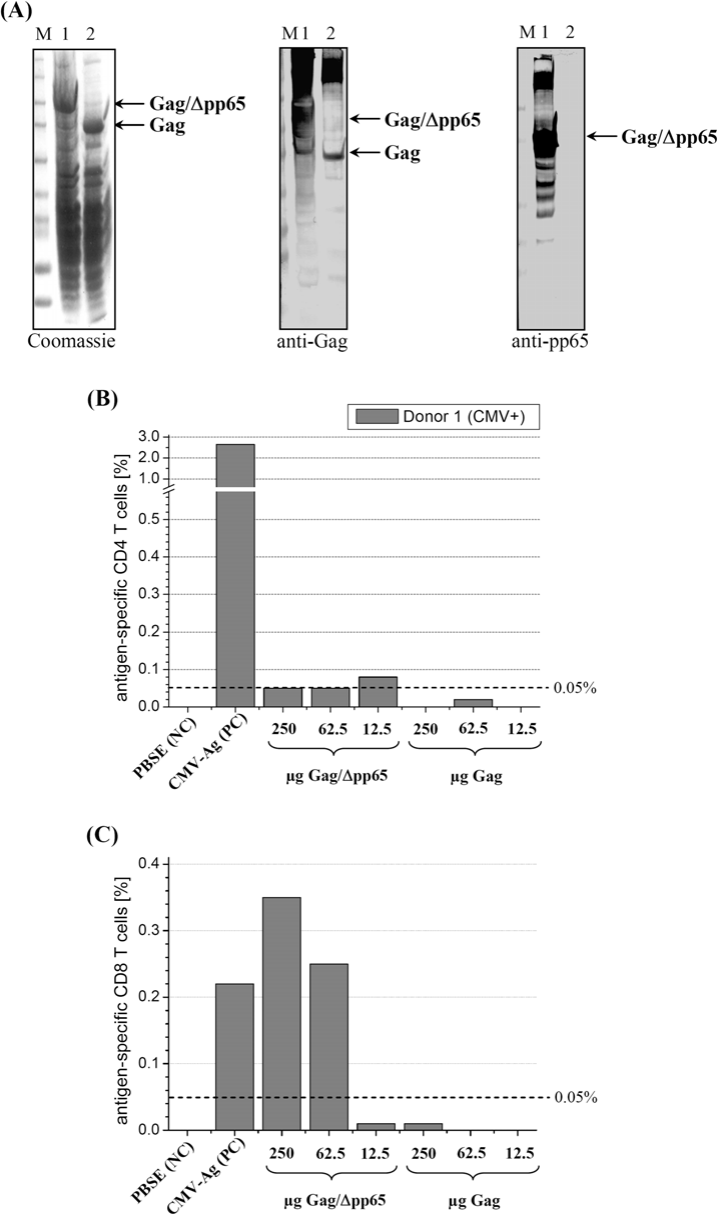
Gag/Δpp65 expressed in yeast assembles into VLP chimeras strongly activating CD8^+^ memory T cells in human whole blood. (A) Western blot of Gag/Δpp65 (lane 1) and Gag (lane 2) expressed in yeast and partially purified as sucrose cushion pellet after ultracentrifugation. Aliquots (20 µl each) of the indicated VLP preparation were subjected to SDS-PAGE followed by Coomassie-Blue staining and western analysis probed with anti-Gag and/or anti-pp65 [M, full range rainbow marker, Amersham]. (B) Frequencies of antigen-specific CD4 and (C) CD8 T cell activation after stimulation by sucrose cushion-purified yeast Gag and Gag/Δpp65 particles. Activated T cells were identified as CD69/IFN-γ double-positive lymphocytes by flow cytometry. Antigen samples were added to whole blood from HCMV seropositive donor 1. A VLP-free sample containing PBSE buffer was included as negative control (NC), a lysate from HCMV-infected fibroblasts served as positive control (PC). The threshold of significant T cell responses (0.05% of counted lymphocytes [15]) is indicated as dashed line.

**Figure 4:**
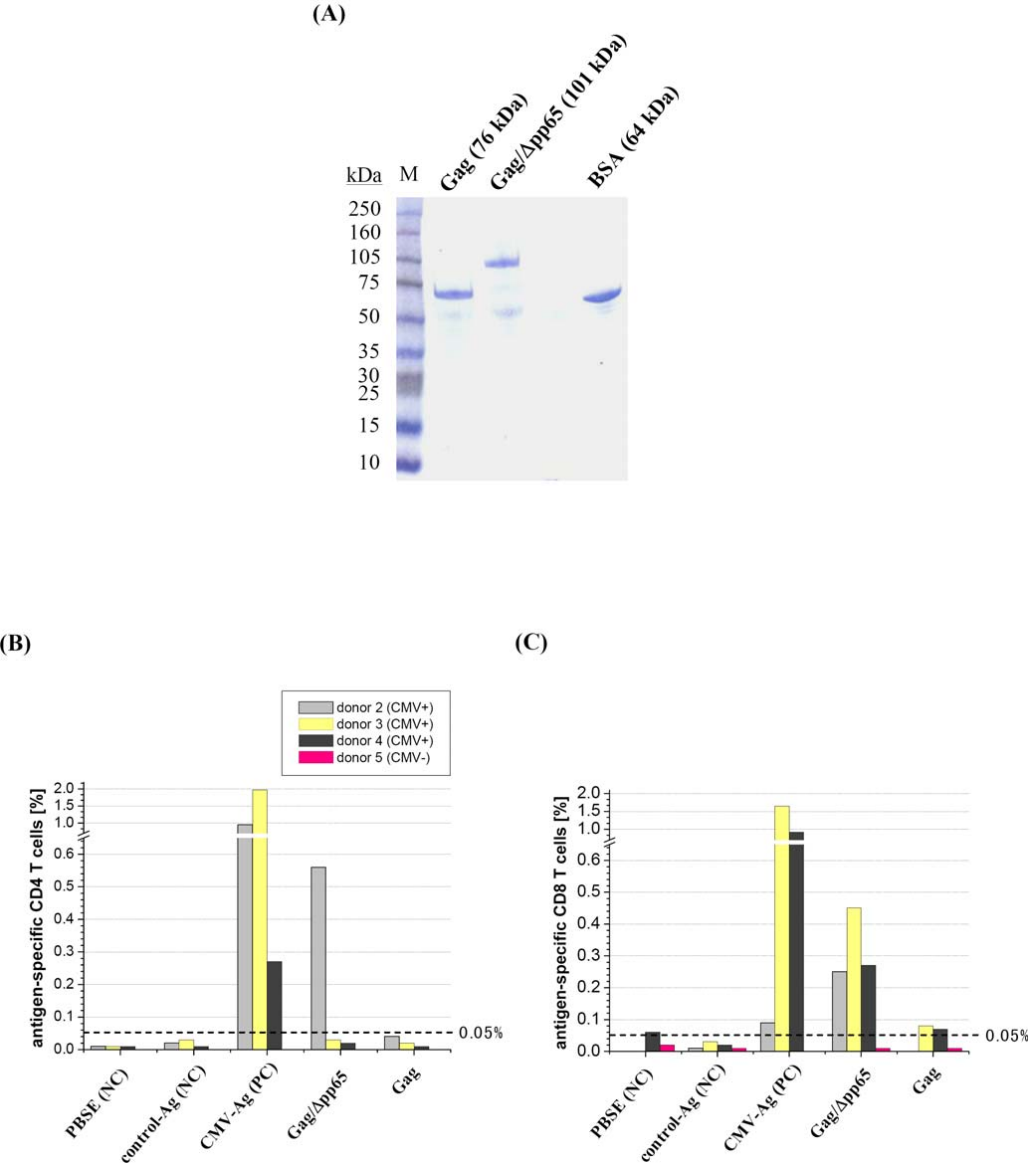
Purified Gag/Δpp65 chimeras induce an extensive human CD8 T cell response. (A) SDS-PAGE and anti-pp65 immunoblot of sucrose gradient purified Gag and Gag/Δpp65 particles expressed and assembled in yeast [Coomassie-Blue staining and BSA (2.7 µg) were used for semi-quantitative signal detection; M, full range rainbow marker, Amersham]. (B and C) Whole blood cells from three HCMV seropositive donors were stimulated by the addition of either Gag or Gag/Δpp65 (5 µg each), and specifically activated CD4 (B) and CD8 (C) T cells were quantified as CD69/IFN-γ double-positive lymphocytes by flow cytometry. A lysate from HCMV-infected fibroblasts served as positive control (PC), whereas a lysate from noninfected fibroblasts, a VLP-free buffer sample as well as blood cells from HCMV seronegative donor 5 were used as negative controls (NC). The threshold of significant T cell responses (0.05% of counted lymphocytes [15]) is indicated as dashed line.

**Figure 5:**
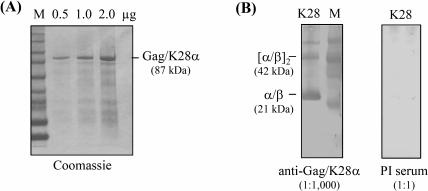
Chimeric Gag/K28α particles displaying the toxic α-subunit of the K28 virus toxin assemble into yeast VLPs that induce an *in vivo* antibody response in rabbit. (A) SDS-PAGE and Coomassie-Blue staining of recombinant Gag/K28α particles expressed and assembled in yeast and purified by sucrose gradient centrifugation. (B) Western analysis of the α/β heterodimeric K28 virus toxin probed with a rabbit polyclonal antiserum raised against chimeric Gag/K28α VLPs assembled in yeast. Positions of the heterodimeric K28 toxin (α/β) and its tetrameric derivative [α/β]_2_ that forms spontaneously under conditions of a non-reducing SDS-PAGE are indicated (PI, pre-immune serum).

Antigenicity of recombinant Gag/Δpp65 particles was determined in an *ex vivo* stimulation assay allowing quantification of HCMV-specific memory T cell responses in human whole blood by FACS analysis [14], [15]. For such an assay, both VLPs (Gag and Gag/Δpp65) were expressed and assembled in yeast and partially purified by centrifugation through a sucrose cushion (Figure 3A). T cell stimulation assays performed on whole blood of a HCMV seropositive donor indicated that pp65-specific CD4^+^ and CD8^+^ T-lymphocytes were strongly activated to maximal frequencies of 2.64% and 0.22% by HCMV positive control antigens, while no immune response was seen in the negative control. Interestingly, cushion-purified Gag/Δpp65 as well as non-modified Gag only poorly activated CD4^+^ cells (<0.1%, Figure 3B), while chimeric Gag/Δpp65 particles induced a pronounced CD8^+^ T-cell response in a dose-dependent manner that was even higher than in the positive control (0.35% versus 0.22%; Figure 3C). In contrast to Gag/Δpp65, unmodified Gag did not significantly activate HCMV-specific CD8^+^ cells, not even at the highest concentration tested (Figure 3C). To demonstrate that the observed CD8^+^ T cell response was caused by the Δpp65 moiety of the chimeric particles, recombinant VLPs were isolated from yeast, purified by sucrose gradient centrifugation and subsequently analyzed by SDS-PAGE and Coomassie-Blue staining. As shown in Figure 4A, gradient-purified VLPs only contained two protein species representing Gag and Gag/Δpp65, thereby demonstrating that both preparations were of high purity (>95%). For T cell stimulation, whole blood of HCMV-seropositive donors was supplemented with 5 µg gradient-purified VLPs; a lysate from HCMV-infected fibroblasts served as positive control, a HCMV-seronegative blood sample as negative control to demonstrate antigen specificity of the immune response. As expected, no T cell response was detectable in the negative control, while a significant CD4^+^ T lymphocyte response was seen in seropositive samples against the positive control antigen (Figure 4B and 4C). In contrast to unmodified Gag, chimeric Gag/Δpp65-VLPs caused a significant activation of CD4^+^ T cells only in donor 2 (Figure 4B). As shown before for cushion-purified particles, gradient-purified Gag/Δpp65 showed significantly elevated frequencies of activated CD8^+^ T cells that were up to 25-fold increased over unmodified Gag (Figure 4C). Qualitatively the same result was obtained by analyzing chimeric VLPs in which the antigenic Δpp65 moiety was expressed on the outer VLP surface by in-frame insertion into surface-exposed loops of Gag immediately upstream of amino acid position S182 and flanked by flexible spacers (Powilleit and Schmitt, unpublished). These data demonstrate that Gag/Δpp65 expressed and assembled into yeast VLPs exposing Δpp65 either inside the particle or at the outer VLP surface, both possess antigenic properties (in particular to activate CD8^+^ memory T cells) that are due to their HCMV-specific Δpp65 moiety. To further investigate the potential of Gag/Δpp65 particles as unique yeast vaccine, we are currently analyzing native Gag/Δpp65 particles in a murine HCMV model of HLA transgenic mice for the induction of a protecting *in vivo* immune response. That antigens exposed inside chimeric yeast VLPs are indeed efficiently processed by immune cells *in vivo* resulting in a humoral immune response was demonstrated by using native Gag/K28α VLPs (assembled in and isolated from a GTXα expressing yeast strain) which induced K28α-specific antibodies in rabbit (Figure 5A and 5B). In addition, since the *in vivo* expression of the α-subunit of the viral α/β toxin K28 is known to be toxic (in particular when expressed in the ER lumen [16]), successful expression of Gag/K28α particles demonstrates that chimeric Gag-VLPs are also suitable for the expression of a *per se* lethal protein.

### Chimeric VLPs as platform for protein expression and purification

**Figure 6:**
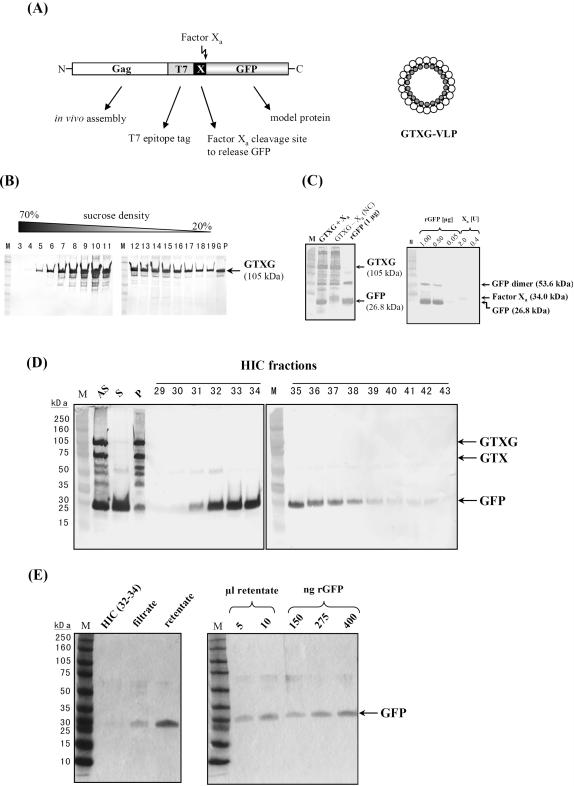
GFP expression and purification *via* recombinant yeast VLPs. (A) schematic outline of a Gag/GFP fusion (GTXG) for *in vivo* VLP assembly and purification of the model protein GFP. The particular function of each domain within the protein fusion is indicated. (B) SDS-PAGE and anti-GFP western analysis of recombinant GTXG particles assembled in yeast and purified by sucrose gradient centrifugation. (C) Release of GFP from GTXG particles by factor X_a_ cleavage [rGFP, recombinant GFP; M, full range rainbow marker, Amersham]. (D) Single-step purification of GFP obtained after factor X_a_ treatment and ammonium sulfate (AS) precipitation (S, supernatant; P, pellet) by hydrophobic interaction chromatography on a HIC column. Samples were separated by SDS-PAGE and probed with anti-GFP and/or anti-T7. (E) Coomassie-Blue staining of pooled GFP-containing HIC fractions after Amicon ultrafiltration through a 10 kDa cut-off membrane [rGFP, recombinant GFP; M, full range rainbow marker, Amersham].

In contrast to monomeric protein fusions, hybrid VLPs are of high molecular weight and can be easily prepared from crude cell extracts by ultracentrifugation [4]. In this context, we exploited Gag as particle-forming carrier to express and purify the green fluorescent protein GFP as model polypeptide. For this purpose, a Gag variant was constructed encoding a 105 kDa protein fusion containing Gag at its N-terminus (to ensure *in vivo* VLP assembly and GFP encapsulation), followed by an 11 amino acid T7 epitope tag (for immunological detection) and a factor X_a_ cleavage site to release mature GFP from Gag (Figure 6A). Yeast transformants expressing such a construct (GTXG) were used for VLP preparation, and western analysis of sucrose gradient fractions revealed that GTXG (105 kDa) assembled into VLPs that showed a sedimentation profile portraying that of natural yeast VLPs (Figure 6B). To check whether the protein fusion is accessible to factor X_a_ cleavage and subsequent release of its GFP moiety, GTXG-VLPs (288 µg protein/ml) were treated with Triton X-100, thereafter incubated with factor X_a_ and subsequently subjected to SDS-PAGE and western analysis. As shown in Figure 6C, detergent-treated GTXG-VLPs were efficiently processed by factor X_a_, liberating two protein moieties from the GTXG precursor (both absent in negative controls) whose calculated molecular weights are consistent with the presence of monomeric (26.8 kDa) and dimeric (53.6 kDa) GFP. In direct support, dimeric GFP was only seen in non-reducing SDS-PAGE and completely disappeared under reducing conditions in the presence of β-mercaptoethanol (data not shown). To investigate the efficiency of preparative GFP purification, detergent-treated GTXG particles (274 µg) were incubated in the presence of factor X_a_, VLP debris was removed by high-spin centrifugation (100.000×g) and the resulting supernatant was treated with Xarrest agarose to eliminate residual endoproteinase. Soluble GFP released from the VLPs was precipitated by the addition of ammonium sulfate, and the resulting pellet and supernatant fraction was subsequently analyzed by SDS-PAGE probed with anti-GFP and anti-T7. By this procedure, monomeric GFP (26.8 kDa) and the larger GTX cleavage fragment (77.8 kDa) were successfully released from the 105 kDa GTXG precursor (Figure 6D). Furthermore, GFP could be purified in a single step by hydrophobic interaction chromatography (HIC) as judged by SDS-PAGE and western analysis (Figure 6D). GFP containing fractions from the HIC column were 100-fold concentrated by Amicon ultrafiltration, and the pooled HIC fractions as well as filtrate and retentate after ultrafiltration were analyzed by SDS-PAGE and Coomassie-Blue staining. As expected, GFP concentration in the retentate was higher than in the pooled HIC fractions or in the filtrate (Figure 6E). Moreover, the preparation was highly pure, only showing two GFP-specific signals on non-reducing SDS gels, a major 26.8 kDa protein representing monomeric GFP and a minor 53.6 kDa species representing dimeric GFP (Figure 6E). Based on the signal intensity after Coomassie-Blue staining, the overall yield of GTXG-derived GFP after sucrose gradient centrifugation and HIC purification was in the range of 0.2 mg purified protein from 1 liter yeast culture (and a density of 5×10^8^ cells/ml).

### VLP chimeras as recyclable biocatalyst

**Figure 7:**
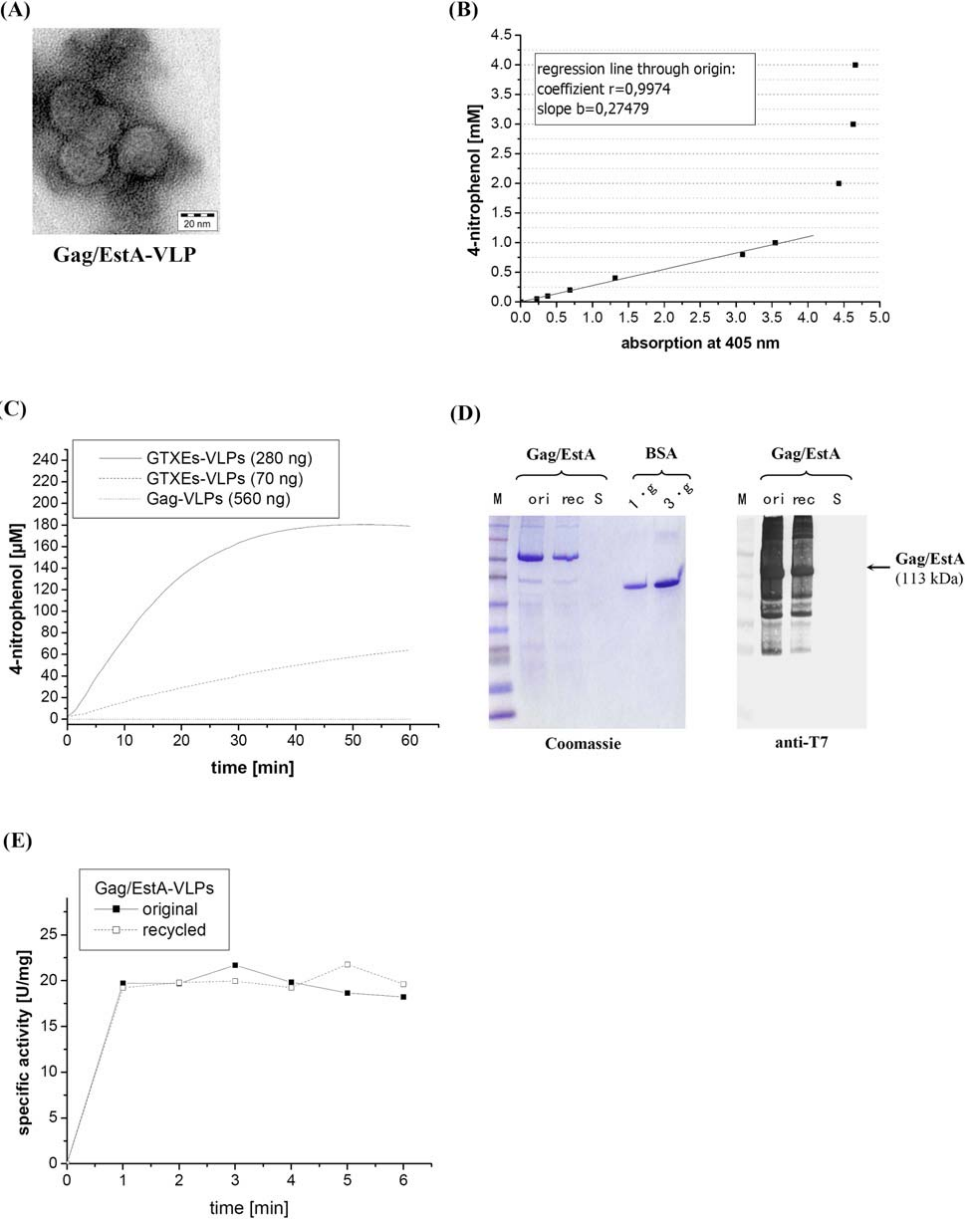
Chimeric yeast VLPs expressing bacterial esterase (EstA) function as recyclable bioreactor and show efficient substrate conversion. (A) Electron micrograph of recombinant Gag/EstA particles prepared from yeast were purified by sucrose gradient centrifugation, negatively stained with uranyl acetate/methyl cellulose and subsequently used for electron microscopy (magnification 340,000). (B) Linear correlation between the 4-nitrophenol concentration of up to 1 mM and its absorption at 405 nm. (C) Kinetics of Gag/EstA-driven hydrolysis of 4-nitrophenylacetate (280 µM) to 4-nitrophenol and acetate at 25°C in PBS_50_ buffer (pH 7.0). (D) Coomassie-Blue staining and western analysis of Gag/EstA particles before and after cata-lysis and recycling by ultracentrifugation. BSA (1 and 3 µg) was used as loading control [M, full range rainbow marker, Amersham]. (E) Specific activity of chimeric Gag/EstA particles before and after recycling.

To demonstrate the flexibility of the viral carrier for the expression of a biotechnologically relevant enzyme, the GFP moiety in GTXG was replaced by the carboxylesterase EstA from *Burkholderia gladioli* [17], the resulting Gag/EstA protein fusion was expressed in yeast and electron microscopy of sucrose gradient-purified Gag/EstA particles confirmed *in vivo* assembly into recombinant VLPs (Figure 7A). To demonstrate esterase activity in the VLP chimeras, gradient-purified Gag/EstA particles were analyzed in an enzyme activity assay using 4-nitrophenylacetate as substrate. Since EstA is located inside the particle, the substrate must pass the capsid pores to be converted into acetate and 4-nitrophenol. Under the assay conditions used, the release of 4-nitrophenol was monitored through its absorption at 405 nm that was shown to be linearly correlated to a concentration of up to 1 mM (regression coefficient = 0.9974; Figure 7B). Based on these parameters, gradient-purified Gag/EstA (70 ng and 280 ng) and non-modified Gag (560 ng) were subsequently analyzed for esterase activity; a VLP-free sample served as negative control to detect autohydrolysis and unspecific breakdown of the ester substrate. In contrast to unmodified VLPs, Gag/EstA chimeras catalyzed the release of 4-nitrophenol and under steady state conditions (reaching reaction equilibrium within 48 min), 67.9% of the initial substrate were enzymatically converted into 4-nitrophenol (Figure 7C).

### Gag/EstA particles allow multiple substrate conversions

To investigate whether particle-associated esterase can be recycled and reused in multiple rounds of substrate conversion, gradient-purified Gag/EstA particles (22.5 µg) were used in an enzyme reaction (5 ml), isolated by ultracentrifugation, subsequently subjected to SDS-PAGE and western analysis, and compared to the same VLP charge prior to substrate conversion. Based on the esterase signal intensity obtained after SDS-PAGE and Coomassie-Blue staining, approximately one-third of the original particle preparation had been recovered in the pellet fraction after a single ultracentrifugation step (Figure 7D). Since Gag/EstA particles were significantly diluted prior to ultracentrifugation, esterase protein remaining in the final supernatant was not detectable in immunoblots. Most interestingly however, catalytic activity of Gag/EstA-VLPs after recovery was not negatively affected and rather resembled EstA activity in the original non-recycled VLPs. Using an equal volume of Gag/EstA particles before and after recycling, absolute esterase activity was three-fold lower in recycled VLPs; however, given that one-third of the initial VLP amount (750 ng) had been recycled, specific esterase activity in the Gag/EstA chimeras before and after recycling was almost identical (Figure 7E), demonstrating that VLP-associated EstA can be recycled and repeatedly used in multiple rounds of enzyme-catalyzed substrate conversion. Most interestingly however, the remarkable efficacy of the VLP-associated enzyme becomes evident when specific esterase activity of Gag/EstA particles (20.8 U mg^−1^ protein) is compared to that after EstA cell surface expression in either yeast (*S. cerevisiae*) or bacteria (*E. coli*): in both cases, esterase activity was significantly lower and ranged from 1.3 to 2.7 U mg^−1^ protein in yeast [18] and 0.001 to 0.023 U mg^−1^ protein in bacteria [19].

## Discussion

Viral expression systems are not only useful in gene transfer experiments, but also in heterologous protein production. In most cases, structural or regulatory elements of animal and human viruses represent the key elements in these systems, restricting their application to higher eukaryotic cells as host. In the present study we engineered the yeast totivirus L-A and demonstrated its feasibility for being used as unique expression system in a lower eukaryotic host. The potential of its capsid as platform for the presentation of immunogens was demonstrated by using the HCMV tegument protein pp65 as model antigen. This structural protein represents the major target of cellular immune response during HCMV infection [20], [21], and also *in vitro* HCMV-infected cells are recognized by 70–90% of cytotoxic T lymphocytes (CTLs) [22]. Besides inducing a strong CD8^+^ T cell response, pp65 can also activate CD4^+^ T cells [23] making it an ideal candidate in developing an HCMV vaccine ensuring both, humoral and cellular immunity.

To analyze recombinant yeast VLPs for their potential as non-replicating particle vaccine, we fused an N-terminally truncated pp65 variant (Δpp65) of HCMV containing immunodominant T cell epitopes to the C-terminus of Gag and showed that it self-assembled into VLP chimeras when expressed in the yeast cell cytosol. Electron microscopy revealed a spheric symmetry of the recombinant particles (Gag/Δpp65) in which the Δpp65 moiety was buried inside the capsid, analogous to Pol in the natural L-A virus [11]. The “*in viro*” localization of Δpp65 either inside or outside the particle was judged in two ways: (i) by immunogold labelling and electron microscopy, and (ii) by analyzing co-sedimentation profiles of Gag/Δpp65 and monoclonal anti-pp65 in a sucrose density gradient (Powilleit and Schmitt, unpublished). In contrast to non-modified (naked) Δpp65 which was only weakly expressed and subject to proteolytic degradation *in vivo*, particle-associated Δpp65 was highly stable and effectively protected against the action of host cell proteases. In an *ex vivo* stimulation assay in which memory T cell stimulation can be quantified and characterized in human whole blood [14], purified Gag/Δpp65 particles - in contrast to non-modified Gag - resulted in a significant activation of CD8^+^ T lymphocytes, while frequencies of activated CD4^+^ helper T lymphocytes (HTLs) were generally low. The same holds true for chimeric VLPs in which the Δpp65 moiety was exposed at the outer VLP surface by insertion into surface-exposed loops of Gag immediately N-terminal to position S182 and flanked by flexible spacer elements (Powilleit and Schmitt, unpublished). In all cases the observed bias in activation of CD8^+^ T lymphocytes by exogenous antigen points to an alternative presentation pathway favouring association of pp65 epitopes with MHC I. In the classical pathway of antigen presentation, peptides derived from exogenous proteins or particles are exposed in complex with MHC II molecules on the cell surface where interaction with complementary T cell receptors leads to an activation of CD4^+^ HTLs. This mechanism is apparently true for the positive control antigen used in this study, a lysate of HCMV-infected fibroblasts containing both soluble as well as virion-associated pp65. In one out of four blood samples, Gag/Δpp65 also induced a CD4 T cell response, indicating that Δpp65 peptides can also be presented in complex with MHC II. Due to the N-terminal truncation in Δpp65, HTL epitopes in pp65 such as peptides 11, 71 and/or 72 are lacking [23], probably attenuating its ability to induce a more frequent CD4^+^ T cell response.

In contrast to MHC II, MHC I-associated presentation of peptides is considered to be restricted to endogenously synthesized proteins, initiating with proteasomal processing in the cytoplasm. Upon targeting on the cell surface, the MHC I/peptide complex can activate CD8^+^ T cells through interaction with the corresponding T cell receptor [24]. More recent studies have indicated that proteins taken up by phagocytosis can also be presented by MHC I molecules, thereby promoting CD8^+^ CTL proliferation [25]. Such alternative antigen presentation (also known as cross-presentation) has been observed in phagocytic cells upon engulfment of bacterial cells or viral particles [26]–[30]. Since recombinant Gag/Δpp65-VLPs share the particulate nature with these antigens, a cross-presentation pathway might also exists for them as well as for pp65 associated to intact HCMV virions (present in the positive control).

Current strategies in HCMV vaccine development imply the application of live, attenuated virus strains, DNA vectors coding for immunodominant HCMV proteins and/or genetically modified carrier viruses [31]. Although these approaches might be well-tolerated and immunogenic, they bear the risk of reconverting to original virulence, inducing anti-DNA antibodies or recombining with the host cell genome [32], [33]. In addition, the production of most of these vaccines in human cell lines is costly, time-consuming and hardly suitable to an industrial scale-up [34]. In contrast, yeast is regarded as safe due to its GRAS status and widely accepted as a profitable host to produce biotechnologically and pharmaceutically relevant proteins [35]. As proof of principle for a yeast vaccine based on recombinant VLP chimeras, we intend to use Gag/Δpp65 particles and HLA transgenic mice in a murine HCMV model to analyze *in vivo* immune responses and to evaluate vaccine potential of chimeric yeast VLPs.

Besides being attractive in vaccine development, the yeast viral expression system described here is also interesting in foreign protein production. This was demonstrated for a gene fusion in which the 3′-end of *gag* was sequentially extended by a T7 epitope, a factor X_a_ cleavage site, and the coding sequence of GFP as model protein. After *in vivo* expression, the protein fusion self-assembled into hybrid yeast VLPs from which GFP could be entirely released from its Gag carrier by factor X_a_ cleavage. Single-step purification *via* hydrophobic-interaction chromatography and subsequent ultrafiltration resulted in a highly pure GFP preparation with an overall yield of 0.2 mg purified and biologically active GFP from 1 liter yeast culture (and a density of 5×10^8^ cells/ml). The overall yield in heterologous GFP production *via* chimeric yeast VLP expression falls within the broad-range levels of GFP fusion protein production which has been shown to range over 3 orders of magnitude, from 4 µg/liter to 4 mg/liter yeast cell culture [36], [37]. Furthermore and in contrast to expression systems based on yeast Ty retrotransposons exposing foreign proteins at the outer VLP surface [38], [39], recombinant Gag particles described here contain their cargo within the inner capsid, thereby effectively preventing proteolytic degradation. Especially for the production of short-lived and unstable proteins (such as pp65 from HCMV), the L-A-derived expression system might be superior as it efficiently protects its cargo from proteolytic degradation in the host cell cytosol. Furthermore, by using the α-subunit of K28 toxin - which is cytotoxic when expressed in yeast [16] - we could demonstrate that recombinant Gag-VLPs are also suitable for the *in vivo* expression of a protein which is *per se* toxic. In addition, Gag was also shown to be effective in the expression of a particle-associated and recyclable biotechnical enzyme, carboxylesterase A from *B. gladioli* [18]. Gag/EstA protein fusions expressed in yeast assembled into VLP chimeras that were catalytically active and effectively converted 4-nitrophenylacetate into 4-nitrophenol and acetate. A hallmark of this VLP-based “bioreactor” is its reusability in multiple substrate conversions without loss in enzyme activity and its overall yield in particle-associated specific esterase activity, being significantly higher than esterase activity after cell surface display in *E. coli* or *S. cerevisiae* [18], [19]. In sum, these data demonstrate the efficiency of the yeast L-A viral expression system in the production and purification of recombinant proteins/enzymes in a particle-associated manner, providing substantial yields of a functional protein in sufficient quality without the need of time-consuming purification procedures. In addition, the ease of fermentation in low-cost media makes *S. cerevisiae* and its chimeric Gag-VLPs attractive for foreign protein production.

## Materials and Methods

### Strains, oligonucleotides and plasmids

*E. coli* strain TOP10 [F^−^ *mcr*A Δ(*mrr-hsd*RMS-*mcr*BC) Φ80*lac*ZΔM15 Δ*lac*X74 *rec*A1 *deo*R *ara*D139 Δ(*ara-leu*)7697 *gal*U *gal*K *rps*L (Str^R^) *end*A1 *nup*G] (Invitrogen) used for plasmid propagation was grown at 37°C in Luria Broth supplemented with 100 µg/ml ampicillin. All plasmids and oligonucleotide primers used in this study are listed in supplementary Table S1 and Table S2, respectively. Target gene amplification was performed using High-Fidelity *Taq* polymerase (Roche) according to the manufacturer's instructions. PCR products were subcloned into pCR®II-TOPO (Invitrogen) and checked by DNA sequencing using primers M13for and/or M13rev (5′-labeled with infra-red dye 800; MWG). For Δ*pp65* amplification, template JW4303 and primers 5′pp65epi+3′CMVepi were used. To obtain plasmid pPGK-Δpp65, the Δ*pp65* fragment was inserted into pPGK via *Eco*RI/*Bam*HI. The *gag*-ORF, amplified using pTIL05 [40] as template and primers 5′L-A ORF1+3′L-A ORF1, was cloned as *Hin*dIII/*Bam*HI fragment into vector pPGK to give pG. To obtain expression plasmids pGAG/Δpp65 and pGTXG, PCR reactions were carried out using the template/primer combinations JW4303/5′CMVepi+3′CMVepi and pUG36/5′T7Xa-GFP+3′GFP, respectively. Upon subcloning, both fragments were inserted as *Sac*I/*Bam*HI fragment into pG. Plasmid yGTXG was constructed by introducing the *Hin*dIII/*Bam*HI GTXG fragment from pGTXG into YEp352. The Xα gene (encoding the α-subunit of killer toxin K28) was amplified using vector pM28-SL [16] as template and primers 5′SpeXal/3′altaaBgl. Subsequently, the 5′-terminal *Spe* I/*Bam* HI fragment was integrated into yGTXG [*Spe* I/*Bam* HI] to give yGTXαΔ. GTXαΔ [*Hin* dIII/*Bam* HI] was then inserted into pPGK, and the GTXα fusion was completed by inserting the 3′-terminal *Bgl* II/*Bam* HI fragment of Xα into the *Bam* HI digested vector pGTXαΔ. For amplification of the *Ce* fragment, template JW4303 and primers 5′pp65epi+3′CMVepi were used. To obtain plasmid pCe, the *Ce* fragment was inserted into pPGK via *Eco* RI/*Bam* HI. The multiple protease-deficient *S. cerevisiae* strain S86c [MATα *ura3-2 his3 pra1 prb2 prc1 cps1* L-0 M-0] represents a heat-cured, virus-free derivative of strain S86 [41] that was employed for the *in vivo* assembly of hybrid VLPs as well as for the expression of soluble (naked) Δpp65 in the yeast cell cytosol. If not otherwise stated, cells were cultivated in YPD at 30°C. Yeast cells were transformed by the lithium acetate method [42] and transformants were selected on synthetic complete medium lacking uracil (Ura-d/o). Since in a yeast super-killer *ski3* mutant (defective in exosome complex components) translation efficacy of the poly(A)^−^ transcript of L-A is more effective and dsRNA copy number is significantly increased [43], [44], a Δ*ski3* variant of strain BY4741 [MAT*a* *his3*Δ1 *leu2*Δ0 *met15*Δ0 *ura3*Δ0 Δ*ski3*] (Euroscarf) was used to prepare natural L-A virions.

### VLP preparation

Transformants of the indicated yeast strain were incubated in 400 ml Ura-d/o at 220 rpm (30°C, in a 1 l-Erlenmeyer flask) to a density of 5×10^7^–5×10^8^ cells/ml, harvested by 10 min centrifugation at 5,000×g (4°C), washed in prechilled H_2_O, thereafter in 1 M sorbitol, and finally resuspended in 50 ml cold PBSES (150 mM NaCl, 10 mM Na_2_HPO_4_ pH 7.4, 10 mM EDTA, 1 M sorbitol). Subsequently, 2-mercaptoethanol (1∶2,000) and 2.5 mg zymolyase 20T (Seikagaku, Japan) were added. Upon 1.5 h incubation at 120 rpm (30°C), spheroplasts were collected by 15 min centrifugation at 500×g (4°C) and washed in cold PBSES. Thereafter, cells were resuspended in 10 ml PBSE (150 mM NaCl, 10 mM Na_2_HPO_4_ pH 7.4, 10 mM EDTA) and disrupted by vortexing seven times for 1 min (with 1 min breaks in between to cool samples on ice) in the presence of 12 g glass beads (0.45–0.55 µm in diameter). The resulting raw extracts were supplemented with 10 ml PBSE and centrifuged at 10,000×g for 1 h (4°C) to sediment glass beads and cell debris. The supernatant was adjusted with PBSE to 23 ml and then layered onto a cushion of 15 ml 45% sucrose. During ultracentrifugation at 69,260×g overnight (4°C; Beckman SW28 rotor) only structures of high molecular weight pass the cushion and form a pellet. Subsequently, the cushion pellet was resuspended in 1 ml PBSE and layered onto a linear density gradient (38 ml) of 20–70% sucrose. Upon further ultracentrifugation at 76,740×g overnight (4°C) the gradient was fractionated into 18–20 fractions (each 2 ml) while the gradient pellet was resuspended in 2 ml PBSE. Aliquots of each fraction were subjected to SDS-PAGE followed by western analysis or Coomassie blue staining. For reisolation of recombinant VLPs, a maximum of 12 fusion protein containing gradient fractions was pooled, supplemented with PBSE to 38 ml, and again ultracentrifuged at 76,740×g overnight (4°C). Finally, the VLP pellet was resuspended in 100–500 µl PBSE. The procedure described above was also used to prepare natural L-A particles from yeast strain BY4741 starting from a 200 ml YPD culture grown to a density of 5×10^8^ cells/ml.

### Rapid extraction (S80 method) and detection of intracellular proteins

Yeast cells from a 1 ml overnight culture were collected by 3 min centrifugation at 8,500×g (20°C) and washed in H_2_O. Upon removal of the supernatant, the pellet was resuspended in the residual liquid and incubated at −80°C for 15 min. Cells were thawed on ice, mixed with 100 µl 3× TT sample buffer (0.15 M Tris/HCl pH 6.8, 12% SDS, 30% glycerol, 0.03% Coomassie Blue R250, 0.6% 2-mercaptoethanol) and heated at 100°C for 5–10 min with occasional vortexing. Thereafter, samples were centrifuged at 17,000×g for 3 min (20°C) to pellet cell debris. Subsequently, the supernatant was transferred into a fresh reaction tube, and 5–20 µl aliquots were separated by SDS-PAGE. Aliquots of the gradient fractions and the cushion and VLP pellets, as well as HIC fractions (20 µl each) were mixed with 10 µl 3× TT sample buffer, boiled for 3 min and subjected to SDS-PAGE. Protein samples were applied onto 7.5% SDS-polyacrylamide gels and run in Tris/Tricine buffer [45]. Upon separation, proteins were either stained with Coomassie Brilliant Blue R250 (Roth) or blotted onto polyvinyl difluoride membranes [46]. Blots were probed with monoclonal anti-pp65 (Novocastra), anti-T7 (Novagen), anti-GFP (Roche) or polyclonal antibodies raised in rabbit against native Gag-VLPs (anti-Gag) or chimeric Gag/K28α particles (anti-Gag/K28α) followed by treatment with an alkaline phosphatase-coupled secondary anti-mouse immunoglobulin (Sigma). For colorimetric signal detection blots were covered with NBT/BCIP solution (Roche) according to the instructions of the manufacturer. Protein concentration was determined by using a bicinchoninic acid assay kit (Sigma). Alternatively, defined amounts of bovine serum albumin (BSA; Sigma) served as standard for semi-quantitative determination of protein concentration after SDS-PAGE and Coomassie Blue staining.

### Transmission electron microscopy

An aliquot of gradient-purified VLPs was layered onto a cupper grid (mesh 300–400; coated with poly-L-lysine) and allowed to bind for 5 min at room temperature. Upon washing three times with 30 µl TBS (150 mM NaCl, 100 mM Tris/HCl pH 7.5) the grid was incubated in uranyl acetate/methyl cellulose (1.8%/0.2%) for 5 min at room temperature (negative staining) before it was slowly dried. For analysis and documentation of VLP samples, a transmission electron microscope type TECNAI G^2^ (FEI) equipped with a MegaView III camera (Olympus) was used.

### Protein processing using factor X_a_ and hydrophobic interaction chromatography (HIC)

To release the GFP moiety from chimeric particles, GTXG-VLPs were prepared from gradient fractions and resuspended in 0.9 ml H_2_O. Upon addition of Triton X-100 (1% final concentration) the sample was rotated overnight at 20°C. Digestion was carried out using 55 mU factor X_a_/µg GTXG protein in a total volume of 4.5 ml at 20°C under rotation for 4 d. To remove residual intact capsid and factor X_a_, the sample was ultracentrifuged at 102,000×g (4°C) for 1 h, and the supernatant was treated with Xarrest agarose (Novagen) according to the manufacturer's instructions. The resulting sample (10 ml) was carefully supplemented with 850 mM ammonium sulfate and subsequently centrifuged at 12,400×g (4°C) for 1 h. The pellet was resuspended in 10 ml H_2_O while 2.5 ml aliquots of the supernatant were applied onto a column of Phenyl Superose HR5/5 (2 ml; Amersham Pharmacia) equilibrated in HIC buffer (pH 7.4) containing 850 mM ammonium sulfate and 100 mM KH_2_PO_4_. The column was washed with the same buffer and bound proteins were eluted in a linear gradient (15 ml) from 850 to 51 mM ammonium sulfate/100 mM KH_2_PO_4_ (pH 7.4). The column was run at 0.5 ml/min, and 1 ml fractions were collected and analyzed by SDS-PAGE.

### Microtiter activity assay

Catalytic activity in esterase-coupled VLPs was determined in microtiter plates (96 flat-bottom wells; Nunc) using an MF reader V2.9-0 (EMS). A stock solution of 80 mM 4-nitrophenylacetate (dissolved in dimethyl sulfoxide) was diluted to the indicated concentrations to serve as substrate solution. To start the reaction, recombinant protein (5 µl) was added to 90 µl PBS_50_ buffer (150 mM NaCl, 50 mM Na_2_HPO_4_, pH 7.0), finally completed with 5 µl substrate solution to give a total volume of 100 µl. The release of 4-nitrophenol was photometrically measured at 405 nm (25°C) in 30 s intervals. Immediately prior to each measurement, the samples were automatically shaken at 600 rpm. The absolute activity [U] of the enzyme was calculated by the ratio of released 4-nitrophenol [µmol] within 1 min, while the specific activity is given by the quotient of absolute activity to the protein amount [U/mg].

### Whole blood assay and flow cytometry

The procedures were performed as breviously described [15]. Briefly, T cell stimulations were carried out using heparinized human blood (450 µl) of four HCMV-seropositive and one HCMV-seronegative European individuals and different amounts of antigen in a standard volume of 45 µl (adjusted with PBSE buffer). Lysates of HCMV-infected and non-infected fibroblasts (CMV-Ag and control-Ag, BioWhittaker) served as controls. To determine the frequency of antigen-specific T cells by flow cytometry, at least 25,000 cells - each of CD4^+^ and CD8^+^ lymphocytes - were analyzed on a FACScan (Becton Dickinson) using the Cellquest software. CD4^+^ and CD8^+^ T cells were identified by gating on the lymphocyte population via cell size and granularity and their high CD4 or CD8 expression level, respectively. Specifically activated T lymphocytes were identified and quantified as CD69 and IFNγ double-positive cells. Due to the observation that negative controls can lead to a minimal activation of T cells [15], the threshold of a significant response was defined to be 0.05% of counted T lymphocytes.

## Supporting Information

Table S1 Origin and properties of plasmids used in this study
(0.04 MB DOC)

Table S2 Sequences of oligonucleotides primers used in this study
(0.05 MB DOC)
