# Supplementary material for: Exploiting the Yeast L-A Viral Capsid for the In Vivo Assembly of Chimeric VLPs as Platform in Vaccine Development and Foreign Protein Expression
Source: PLoS One. 2007 May 2;2(5):e415. doi: 10.1371/journal.pone.0000415 (PMC1853235; doi:10.1371/journal.pone.0000415)
Supplement: Table S1 — Origin and properties of plasmids used in this study (0.04 MB DOC) [file pone.0000415.s001.doc]

**Table S1.** Origin and properties of plasmids used in this study

| **Plasmid** | **Origin/Reference** | **Properties** |
| --- | --- | --- |
| pCR®II-TOPO | Invitrogen (The Netherlands) | TA cloning vector [Amp^R^/Kan^R^] |
| YEp352 | Hill et al. [47] | 2µ yeast/*E. coli* shuttle multi-copy vector [*URA3*/Amp^R^] used for subcloning |
| JW4303 | A. Meyerhans, Homburg/Germany | *E. coli* vector [Amp^R^] containing the *pp65* gene of human cytomegalovirus (HCMV) |
| pUG36 | J. Hegemann,  Düsseldorf/Germany | 2µ yeast/*E. coli* shuttle vector [Amp^R^] encoding the yeast enhanced GFP variant yEGFP3 |
| pFB-EST | F. Breinig,  Saarbrücken/Germany | *E. coli* vector [Amp^R^] containing *EstA* from *Burkholderia gladioli*. The single *Sal*I-restriction site within *EstA* was destroyed by site-directed mutagenesis |
| pTIL05 | Icho and Wickner [40] | Bluescript vector [Amp^R^] harbouring a cDNA of the L-A variant “HNB” |
| pPGK | Kang et al. [48] | 2µ yeast/*E. coli* shuttle multi-copy expression vector [*URA3*/Amp^R^] containing the yeast phosphoglycerate kinase (*PGK*) promoter and terminator |
| yGTXG,  yGTXα, yGTXEs | This study | 2µ yeast vector [*URA3*/Amp^R^] harbouring the gene fusions *GTXG*, *GTXα* and *GTXEs* |
| pG vector series  (pGAG/∆pp65, pPGK-∆pp65, pGTXEs, pGTXG, pGTXα) | This study | 2µ yeast vector [*URA3*/Amp^R^] expressing various L-A *gag* fusions from the yeast *PGK1* promoter (constructs *GAG/*∆*pp65*, ∆*pp65*, *GTXEs*, *GTXG* and *GTXα* respectively) |
