# Supplementary material for: Exploiting the Yeast L-A Viral Capsid for the In Vivo Assembly of Chimeric VLPs as Platform in Vaccine Development and Foreign Protein Expression
Source: PLoS One. 2007 May 2;2(5):e415. doi: 10.1371/journal.pone.0000415 (PMC1853235; doi:10.1371/journal.pone.0000415)
Supplement: Table S2 — Sequences of oligonucleotides primers used in this study (0.05 MB DOC) [file pone.0000415.s002.doc]

**Table S2.** Sequences of oligonucleotides primers used in this study

| **Primer** | **Sequence** |
| --- | --- |
| 5’L-A ORF1 | 5’-ggg AAG CTT *atg* cta aga ttt gtt act aaa aac tc-3’ |
| 3’L-A ORF1 | 5’-tcc gga tcc *tta* gag ctc tac taa aac att gtc cg-3’ |
| 5’CMVepi | 5’-g GAG CTC ata tcc gta ctg ggt ccc att tcg ggg cac gta cag cgc ggg cct cag tac agc gag-3’ |
| 5’pp65epi | 5’-g GAA TTC *atg* ata tcc gta ctg ggt ccc att tcg g-3’ |
| 3’CMVepi | 5’-g GGA TCC *tta* cac gtt gat gct ggg gat gtt cag cat acc tcg gtg ctt ttt ggg cgt caa gg-3’ |
| 5’T7Xa-GFP | 5’-a GAG CTC *atg* gct agc atg act ggt gga cag caa atg ggt ACT AGT att gag gga cgc *atg* tct aaa ggt gaa gaa tta ttc act gg-3’ |
| 3’GFP | 5’-c ccg GGA TCC *tta* ttt gta caa ttc atc cat acc atg g |
| 5’SpeXal | 5’-a ACT AGT att gag gga cgc gac ttc agt gct gct act tgc gta ctg at-3’ |
| 3’altaaBgl | 5’-a AGA TCT tta cga ttg tat att ctc tgc gtt ttc agt ctt ac-3’ |
| 5’XEs | 5’-ACT AGT att gag gga cgc *atg* gtc cag ctc cat atg ggc ggc ggt-3’ |
| 3’EsEnd | 5’-GGA TCC *tta* ctt ggt gac gcc ggc cgc cgc gat c-3’ |
| M13rev (5’IRD800) | 5’-gag cgg ata aca att tca cac agg-3’ |
